# Efferocytosis: unifying pathogenic hub in metabolic disorders—mechanistic landscapes, targeted therapies and translational bottlenecks

**DOI:** 10.3389/fimmu.2026.1886016

**Published:** 2026-07-27

**Authors:** Dongze Li, Yao Huang, Xuanqin Chen, Li Zhang, Qiming Gong, Qifu Li, Yu Li, Yong Xu, Wei Huang

**Affiliations:** 1 Department of Endocrinology and Metabolism, The Affiliated Hospital of Southwest Medical University, Luzhou, Sichuan, China; 2 Sichuan-Chongqing Joint Key Laboratory of Metabolic Vascular Diseases, Luzhou, Sichuan, China; 3 Metabolic Vascular Disease Key Laboratory of Sichuan Province, Luzhou, Sichuan, China; 4 Sichuan Clinical Research Center for Nephropathy, Luzhou, Sichuan, China; 5 Department of Gastroenterology, The Affiliated Hospital of Southwest Medical University, Luzhou, Sichuan, China; 6 Department of Burn and Plastic Surgery, The Affiliated Hospital of Southwest Medical University, Luzhou, Sichuan, China; 7 Chinese Academy of Sciences (CAS) Key Laboratory of Nutrition and Metabolism, Shanghai Institute of Nutrition and Health, University of Chinese Academy of Sciences, Chinese Academy of Sciences, Shanghai, China

**Keywords:** clinical translation, efferocytosis, metabolic disorders, metaflammation, phagocyte

## Abstract

Metabolic disorders, such as obesity, diabetes mellitus, metabolic dysfunction-associated steatotic liver disease, and atherosclerosis, collectively pose a severe global public health crisis. Systemic chronic low-grade inflammation caused by metabolic stress, namely metaflammation, is universally present in metabolic disorders. This pathological trait drives causal interactions and synergistic deterioration among distinct metabolic disorders, and cannot be efficiently alleviated by conventional clinical anti-inflammatory drugs. Efferocytosis refers to the programmed clearance of apoptotic cells mediated by phagocytes such as macrophages. It is indispensable for maintaining innate immune homeostasis, facilitating the resolution of metaflammation, and balancing metabolic microenvironments. This review comprehensively outlines the core characteristics and molecular mechanisms of efferocytosis, illustrates its regulatory networks in multiple metabolic disorders, concludes current therapeutic strategies targeting efferocytosis for metabolic disorders, and discusses the controversies and translational challenges of relevant interventions. This review systemically elucidates the shared core pathological mechanisms of defective efferocytosis underlying the interactive and progressive progression of multiple metabolic disorders, lays a theoretical foundation for the development of early diagnostic biomarkers and novel immune-targeted intervention strategies for metaflammation, and provides evidence-based rationale for translational research and clinical practice of related metabolic disorders.

## 1 Introduction

Metabolic disorders encompass a highly heterogeneous spectrum of chronic conditions marked by disruptions in nutrient metabolism and the regulation of energy homeostasis. Among these, obesity, diabetes mellitus (DM), and metabolic dysfunction-associated steatotic liver disease (MASLD) represent the most prevalent and clinically impactful entities (1). The latest released data from the Global Burden of Disease (GBD) study indicate that the worldwide prevalence of metabolic disorders has risen to affect one-third of the global population, these diseases contribute to approximately 18 million deaths annually, imposing an enormous socioeconomic and healthcare burden worldwide (2, 3). Notably, all these metabolic disorders share chronic systemic low-grade inflammation, namely metaflammation, as a common pathological hallmark (4, 5). However, conventional clinical anti-inflammatory agents such as nonsteroidal anti-inflammatory drugs (NSAIDs) show limited therapeutic effects against metaflammation caused by metabolic disorders.

The programmed clearance of apoptotic cells—executed by phagocytes such as macrophages via recognition, engulfment, and degradation—represents a pivotal mechanism by which the innate immune system maintains tissue homeostasis and defends against chronic inflammatory injury (6). As early as 1882, Metchnikoff first observed migratory phagocytes clearing apoptotic cells during embryonic development and metamorphosis in starfish larvae. Later, in 2003, Aimée M. deCathelineau and colleagues formally designated this process “efferocytosis”, a term derived from the Latin *efferre*, meaning “to carry to the grave” (7, 8). Notably, this mechanism extends far beyond simple cellular waste clearance. On the one hand, efferocytosis prevents apoptotic cells from undergoing secondary necrosis and blocks the release of pro-inflammatory intracellular contents. On the other hand, it triggers robust pro-resolving signaling by promoting phagocytes to secrete anti-inflammatory mediators, thereby facilitating tissue repair (9, 10). Dysregulated efferocytosis is firmly established to be tightly linked to the progression of multiple chronic inflammatory disorders, including autoimmune diseases, respiratory diseases such as asthma, and neurodegenerative diseases (11–15). Furthermore, the pivotal role of efferocytosis in metabolic disorders and metaflammation has garnered increasing attention.

To date, multiple reviews have summarized the mechanisms underlying efferocytosis in DM and atherosclerosis (16–19). Nevertheless, existing studies are still confined to specific disease types and fail to systematically integrate the latest advances in other research fields related to metabolic disorders as well as their value for clinical translation. This review addresses this crucial knowledge gap through a comprehensive, integrated analysis. We outline the characteristics and molecular mechanisms underlying efferocytosis. We then elaborate on the reciprocal crosstalk between defective efferocytosis and metabolic disorders—encompassing obesity, MASLD, atherosclerosis, DM, and diabetic microvascular complications. Finally, we summarize therapeutic strategies targeting efferocytosis for metabolic disorders, alongside key controversies and unresolved challenges in clinical translation. This review aims to define efferocytosis dysfunction as a shared core pathogenic mechanism underlying metabolic disorders, and clarify its potential as a universal therapeutic target as well as its prospects for clinical translation across these diseases.

## 2 The characteristics of efferocytosis

Efferocytosis is an active, efficient, and evolutionarily highly conserved biological process (20). Under homeostatic conditions, apoptosis represents the predominant form of cell death (21). Approximately 10 billion to 100 billion apoptotic cells are generated in the human body each day, encompassing parenchymal cells as well as neutrophils, lymphocytes, and other infiltrating immune cells that complete their functional roles during inflammatory resolution (22). These apoptotic cells are rapidly cleared via efferocytosis in a non-inflammatory, immunologically silent manner prior to plasma membrane rupture. Accordingly, apoptotic cells are barely detectable even in tissues with high cellular turnover rates. This cellular “homeostatic clearance” mechanism not only facilitates rapid tissue turnover but also underpins tissue remodeling and the maintenance of physiological homeostasis.

**Figure 1:**
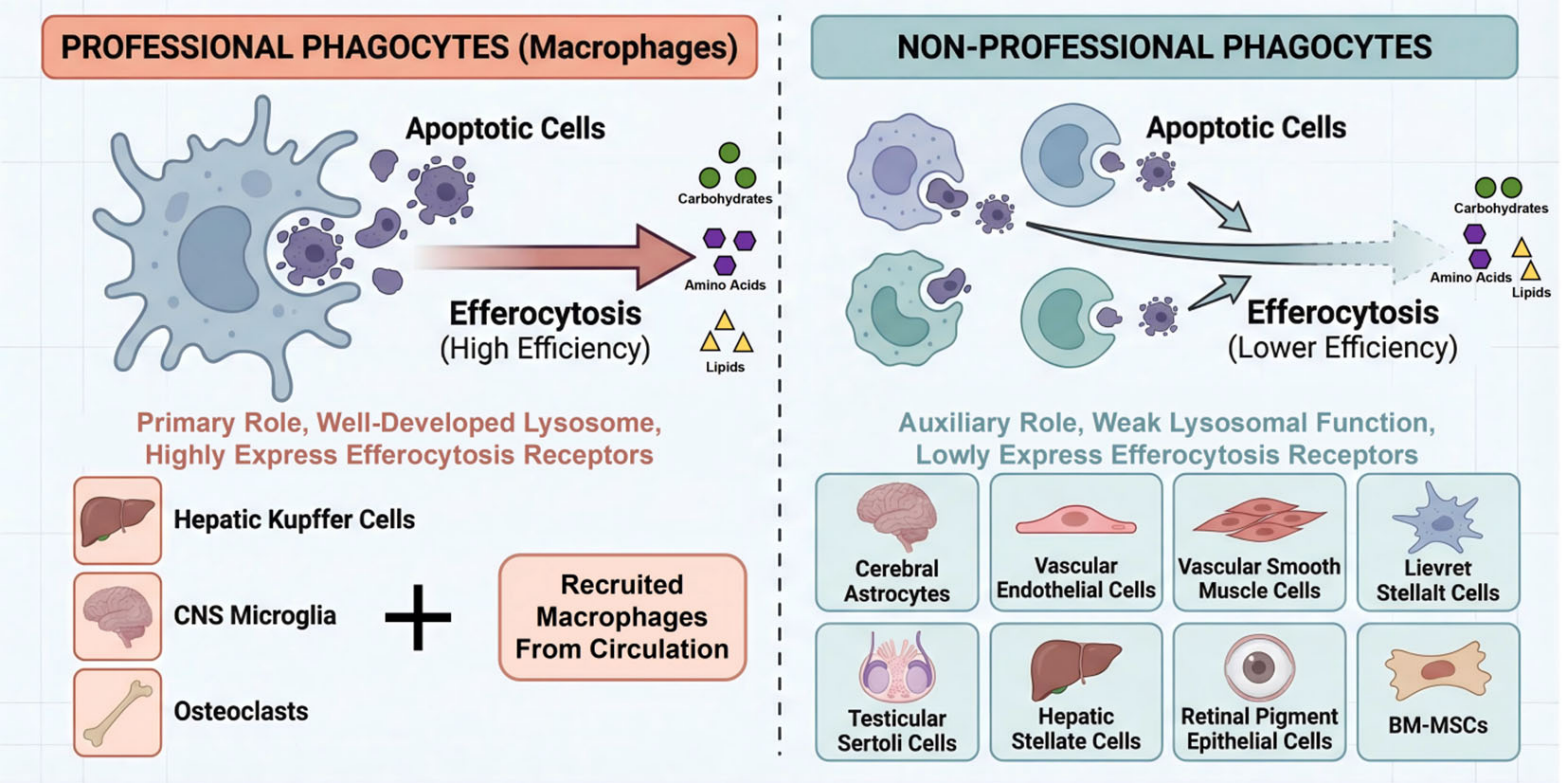
The phagocytes in efferocytosis.

Tissue-resident or infiltrating macrophages act as the primary professional phagocytes responsible for mediating efferocytosis. Owing to their well-developed lysosomal system and high expression of cell-surface efferocytosis receptors, they exhibit significantly stronger phagocytic capacity compared with other cell populations. Representative tissue-resident subsets include hepatic Kupffer cells, central nervous system microglia, and osteoclasts within bone tissue (23, 24). Through strategic tissue distribution, these macrophages continuously surveil the local microenvironment and efficiently recognize and clear apoptotic cells alongside cellular debris. Nevertheless, the removal of the massive number of apoptotic cells *in vivo* is not exclusively performed by macrophages. Under conditions of elevated cellular turnover or tissue injury, multiple non-professional phagocytes also exert critical auxiliary roles in efferocytosis. Such cell populations include cerebral astrocytes, vascular endothelial and smooth muscle cells, testicular Sertoli cells, hepatic stellate cells, retinal pigment epithelial cells, and bone marrow mesenchymal stem cells (BM-MSCs), though their phagocytic efficiency remains markedly lower than that of macrophages (25–29) (**Figure 1**).

Professional phagocytes involved in efferocytosis—chiefly represented by macrophages, such as hepatic Kupffer cells, central nervous system microglia, osteoclasts, and circulating recruited macrophages—act as the primary executors of efferocytosis. These cells are distinguished by a well-developed lysosomal system, abundant expression of efferocytosis-related receptors, and high efferocytic efficiency. By contrast, non-professional phagocytes, including cerebral astrocytes, vascular endothelial cells, vascular smooth muscle cells, hepatic stellate cells, and other tissue-resident cells, only play an auxiliary role in apoptotic cell clearance. Relative to professional phagocytes, they display impaired lysosomal function, low expression of efferocytic receptors, and lower efferocytic efficiency.

## 3 The molecular process of efferocytosis

**Figure 2:**
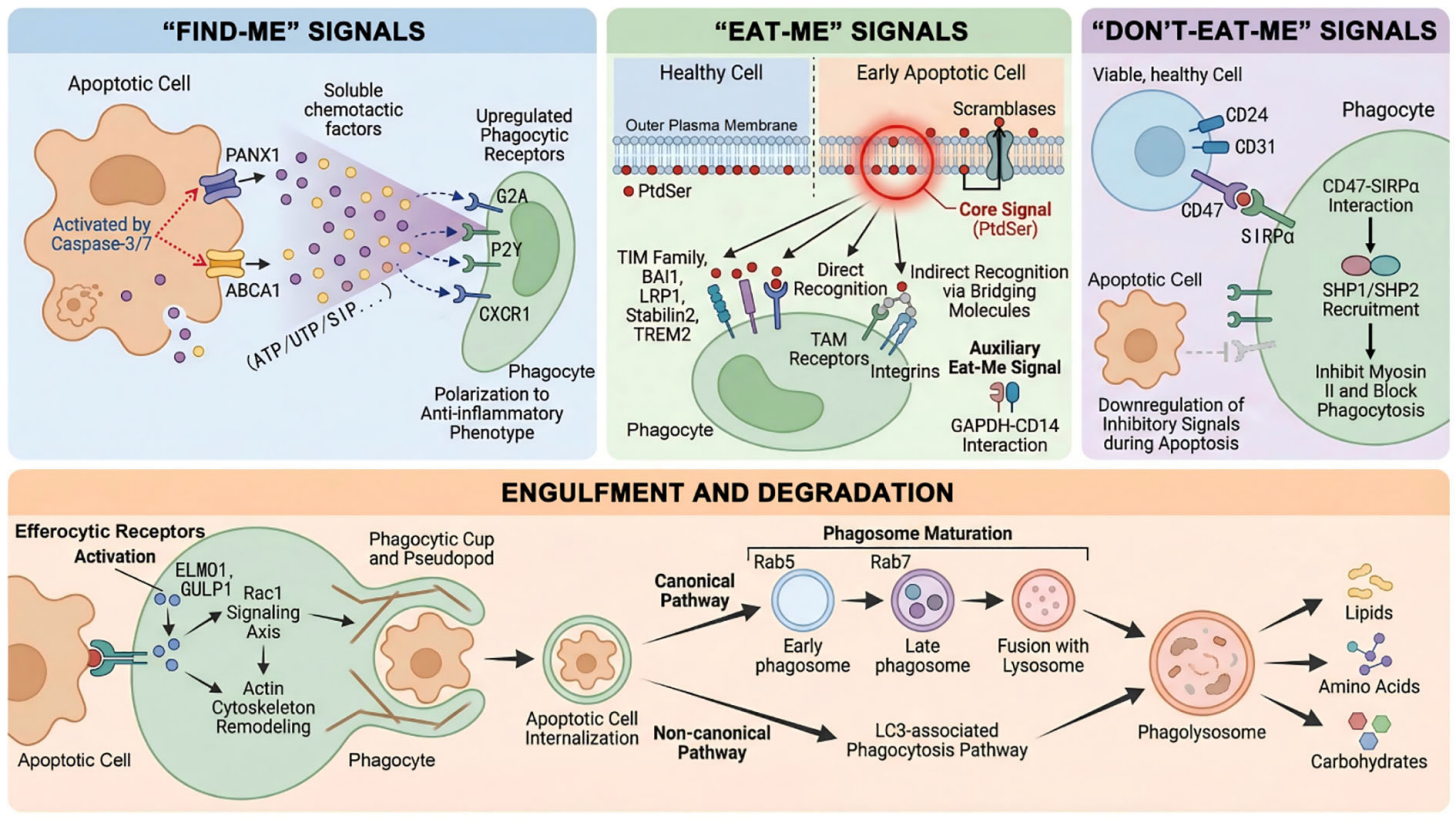
The molecular biological process of efferocytosis.

Unlike conventional phagocytosis which often triggers inflammatory responses, efferocytosis proceeds as a precisely coordinated, dynamic multi-step signaling cascade (30). This process encompasses four sequential stages: sensing and targeting apoptotic cells, specific recognition of apoptotic surface ligands by phagocytic receptors, engulfment of dying cells, and degradative processing of cellular contents. Each phase is governed by distinct molecular signals and receptors, ensuring high efficiency alongside immunologically silent phenotype (31)(**Figure 2**).

Efferocytosis is a core biological process by which phagocytes efficiently clear apoptotic cells. The complete process can be divided into four key stages: 1. The release of “Find-Me” signals: Apoptotic cells release chemotactic factors such as ATP/UTP and S1P through channels including PANX1 and ABCA1, recruiting phagocytes and inducing their polarization to an anti-inflammatory phenotype. 2. The exposure of “Eat-Me” signals: In early apoptotic cells, scramblases mediate the externalization of PtdSer. This signal is directly recognized by receptors such as the TIM family and BAI1, or indirectly binds to TAM receptors and integrins on phagocytes via bridging molecules, thereby initiating phagocytosis. 3. “Don’t-Eat-Me” signals: Viable healthy cells highly express CD47, which binds to SIRPα on phagocytes and activates SHP1/SHP2 to inhibit phagocytosis. Apoptotic cells downregulate this inhibitory signal, releasing the phagocytic block. 4. Engulfment and degradation: Phagocytes initiate actin cytoskeleton remodeling via the ELMO1/GULP1-Rac1 axis, form phagocytic cups to engulf apoptotic cells, and then fuse with lysosomes to form phagolysosomes through either the canonical pathway (Rab5/Rab7-mediated phagosome maturation) or the non-canonical pathway (LC3-associated phagocytosis). Finally, apoptotic cells are degraded into nutrients such as lipids, amino acids, and carbohydrates, maintaining tissue homeostasis.

### 3.1 “Find-Me” signals

Efferocytosis is initiated prior to physical contact between phagocytes and apoptotic cells, beginning with the “Find-Me” phase. Apoptotic cells actively signal their presence by secreting soluble “Find-Me” cues into the surrounding microenvironment. These chemotactic signals are predominantly released via Pannexin 1 (PANX1) and ATP-binding cassette transporter A1 (ABCA1), two channels activated by caspase-3/7. This process establishes chemical gradients that guide phagocyte migration toward apoptotic sites (32, 33). “Find-Me” signals exhibit substantial molecular diversity, encompassing nucleotides including adenosine triphosphate (ATP) and uridine triphosphate (UTP); lipids such as lysophosphatidic acid (LPA), lysophosphatidylcholine (LPC), and sphingosine-1-phosphate (S1P); as well as chemokines represented by C-X3-C motif chemokine ligand 1 (CX3CL1) (34–38). These soluble factors exert multifunctional effects: they act as chemoattractants to guide phagocyte migration toward apoptotic cells, modulate cytoskeletal rearrangement within phagocytes, and upregulate the expression of phagocytic receptors such as CD11b and αvβ3 integrins, thereby priming phagocytes for subsequent engulfment (39, 40). Furthermore, “Find-Me” signals can polarize both infiltrating and tissue-resident phagocytes toward an anti-inflammatory phenotype, thereby maintaining the immunologically silent nature of efferocytosis (28). Phagocytes possess a diverse panel of receptors, including the ATP/UTP receptor P2Y, the CX3CL1 receptor CX3CR1, and the LPC receptor G2A. These enable phagocytes to sense “Find-Me” cues released by apoptotic cells and undergo targeted directional migration (41–44). This initial step is significant for enabling rapid localization of apoptotic cells and minimizing their retention within tissues.

### 3.2 “Eat-Me” signals

When phagocytes migrate to the vicinity of apoptotic cells, they must precisely discriminate target cells from adjacent viable healthy counterparts. This “recognition and screening” phase is governed by intricate interactions between phagocytic “Eat-Me” signals exposed on the apoptotic cell surface. Phosphatidylserine (PtdSer) is the most abundant negatively charged phospholipid within eukaryotic cell membranes. As a membrane-anchored molecule, PtdSer serves as an evolutionarily conserved, well-characterized core “Eat-Me” signal (45, 46). In healthy cells, PtdSer is strictly confined to the inner plasma membrane by ATP-dependent phospholipid translocases (flippases). During early apoptosis, Caspase-mediated inactivation of flippases alongside activation of Ca^2+^-dependent scramblases such as Xkr8 promotes rapid PtdSer externalization (47). Notably, a threshold level of surface-exposed PtdSer is required to initiate engulfment (48).

The exposed PtdSer is recognized by phagocytes via two primary mechanisms. First, it directly binds to surface-expressed PtdSer receptors on phagocytes. Well-characterized recognition receptors include TIM family members (e.g., TIM-1/4), alongside BAI1, LRP1, and Stabilin-2 (49–52). It must be emphasized that these PtdSer receptors exert functions beyond apoptotic cell clearance. TIM-1 and LRP1 selectively bind oxidized low-density lipoproteins (ox-LDL) and fatty acids, respectively, thereby participating in lipid metabolism and triggering anti-inflammatory responses within phagocytes (53–56). TREM2 is a unique transmembrane receptor expressed on the surface of phagocytes. Although it lacks a canonical PtdSer-binding domain, it can still act as a sensor for the lipid microenvironment surrounding apoptotic cells and indirectly participate in PtdSer-dependent phagocytosis. TREM2 recognizes a diverse array of lipid ligands on the plasma membrane of apoptotic cells, including apolipoprotein E (APOE), apolipoprotein J (APOJ), and related lipid complexes. It colocalizes with externally exposed PtdSer on apoptotic cells and synergistically amplifies phagocytic signaling cascades (57, 58).

A prevalent alternative mechanism underlying “Eat-Me” signal recognition relies on indirect binding mediated by soluble bridging molecules secreted by phagocytes. These intermediates concurrently interact with PtdSer on apoptotic cells and specific receptors expressed on phagocytes. Representative bridging factors include secreted proteins such as Gas6 and Protein S, which couple PtdSer to the TAM family of tyrosine kinase receptors—namely Tyro3, Axl, and MerTK (59); as well as MFG-E8, DEL-1 and CCN1, which can connect PtdSer to αvβ3/αvβ5 integrins (60–62). Beyond PtdSer, multiple complementary “Eat-Me” signals provide redundant safeguards to ensure the specificity and accuracy of phagocytic recognition (63). For instance, a distinct surface isoform of GAPDH expressed on apoptotic cells mediates “Eat-Me” signaling via interaction with the phagocytic receptor CD14 on target phagocytes (64). Such redundancy and diversity within these recognition systems maintain the robustness and efficiency of apoptotic clearance across distinct tissues and physiological contexts.

### 3.3 “Don’t-Eat-Me” signals

Counterbalancing the intricate, dynamically regulated network of parallel “Eat-Me” signaling cascades is the “Don’t-Eat-Me” repertoire expressed by viable cells. These two signaling systems act in mutual restraint, thereby preventing healthy cells from aberrant recognition and unintended engulfment by phagocytes (65). A canonical example of this inhibitory signaling cascade lies in the interaction between CD47—ubiquitously expressed on all viable cells—and its phagocytic receptor signal regulatory protein α (SIRPα). This potent anti-phagocytic effect is highly conserved across biological systems (66). When surface-expressed CD47 on viable cells binds to phagocytic SIRPα, it triggers tyrosine phosphorylation within the cytoplasmic domain of SIRPα, which further recruits and activates the phosphatases SHP-1 and SHP-2 in phagocytes. These phosphatases subsequently suppress all pro phagocytic signaling cascades through the dephosphorylation of non-muscle myosin II, a core cytoskeletal component. This mechanism prevents the erroneous engulfment of viable cells even with transient PtdSer exposure, thereby establishing a self-recognition checkpoint *in vivo (*67). These “Don’t-Eat-Me” signals are typically downregulated or masked in apoptotic cells, enabling “Eat-Me” cues to dominate and thereby initiate efferocytosis (68).

Beyond CD47, additional surface molecules on viable cells exert analogous “Don’t-Eat-Me” signaling to avert aberrant clearance of healthy cells. For instance, CD24 on viable cells engages Siglec-10 on macrophages to mediate anti-phagocytic effects (69, 70). Moreover, CD31 on viable cells also transmits inhibitory “Don’t-Eat-Me” cues that prevent phagocytes from engulfing healthy cells, it also facilitates detachment of viable cells from phagocyte surfaces, thereby further sustaining tissue and cellular homeostasis (71).

### 3.4 Engulfment and degradation

Upon recognition of apoptotic cells via phagocyte surface receptors, intracellular signaling cascades are activated to ultimately mediate physical engulfment. This process relies on robust cytoskeletal remodeling, driven predominantly by coordinated Rac1 GTPase activation through the engulfment adaptor GULP1 and the ELMO1/DOCK180 guanine nucleotide exchange factor (GEF) complex (72, 73). By modulating actin filament polymerization, the aforementioned signaling cascade facilitates phagocytic cup formation and pseudopod extension, thereby enclosing and internalizing apoptotic target cells (74). Following the uptake of multiple apoptotic cells, phagocytes dynamically adjust their volume and surface area prior to initiating the intracellular degradation program for engulfed cargos.

Degradation of apoptotic cells proceeds through both canonical phagocytic routes and parallel non-canonical pathways. In the canonical pathway, nascent early phagosomes gradually mature into late phagosomes—a process marked by the small GTPase switch from Rab5 to Rab7—and ultimately fuse with lysosomes to form phagolysosomes. Phagosome maturation additionally requires the recycling regulator Rab17, which diverts apoptotic cellular contents away from antigen-presenting compartments (75). Within the acidic lysosomal microenvironment enriched with hydrolytic enzymes, apoptotic cellular contents are degraded into fundamental metabolic units (including lipids, amino acids, and carbohydrates). In contrast, the non-canonical pathway is exemplified by LC3-associated phagocytosis—a specialized autophagy-related mechanism—that accelerates phagolysosome maturation to enable more rapid and efficient degradation (76). Notably, the degradation of apoptotic cells does not mark the terminal stage of the engulfment process. Metabolites derived from apoptotic cells are not merely eliminated; instead, they integrate into the metabolic pathways of phagocytes to facilitate material recycling. These metabolites can also drive metabolic reprogramming in macrophages, sustaining their robust phagocytic capacity (77).

## 4 Efferocytosis in metabolic disorders

In the progression of metabolic disorders such as obesity and diabetes, persistent glucolipotoxicity induces the accumulation of advanced glycation end products (AGEs), aggravates oxidative stress and inflammatory responses, these collectively constructs intricate pathological tissue microenvironment. This pathological microenvironment directly impairs phagocytic receptor signaling and metabolic sensing functions in both tissue resident and infiltrating phagocytes, leading to disrupted efferocytosis and the induction of pro-inflammatory responses. Of note, emerging evidence suggests that defective efferocytosis is not merely a secondary outcome of metabolic stress, but may also represent a shared central pathological amplifier underlying the progression of multiple metabolic disorders.

### 4.1 Obesity

Obesity is a chronic progressive metabolic disorder characterized pathologically by excessive proliferation and expansion of adipose tissue, and it has evolved into a major global public health concern (78). Sustained lipotoxic accumulation triggered by obesity acts as a critical upstream metabolic stressor, driving robust production of reactive oxygen species (ROS) and diverse pro-inflammatory mediators. These molecular signals further recruit immune cells such as macrophages to infiltrate the microenvironment of adipose and other tissues, while simultaneously blunting immune cell-governed pro-resolving inflammatory programs. Long-term blockade of inflammatory clearance cascades progressively exacerbates systemic insulin resistance, ultimately facilitating the onset and progression of DM, MASLD, and other metabolic complications (79, 80).

**Figure 3:**
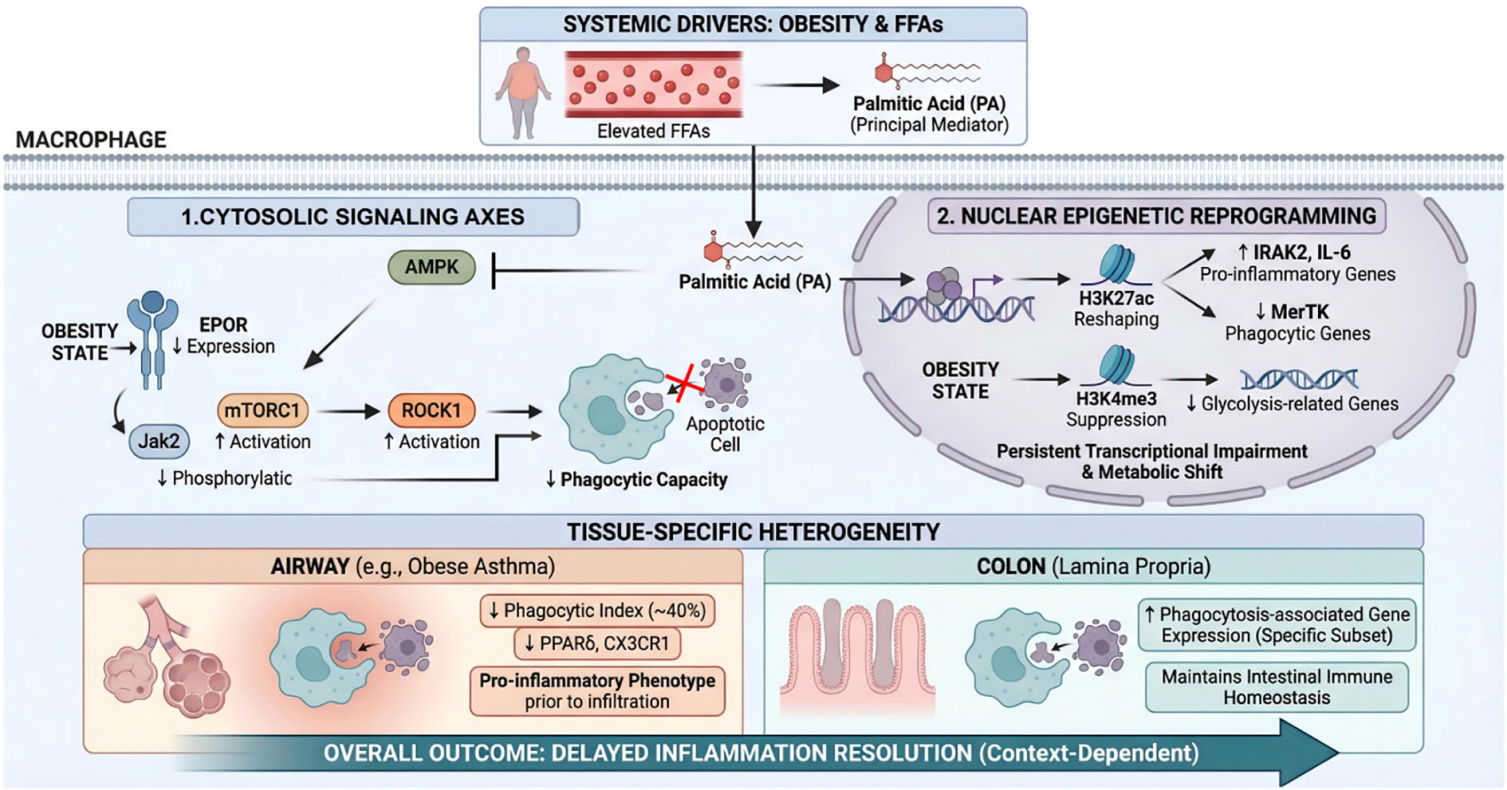
Obesity-induced changes in macrophage efferocytic function.

Cumulative preclinical studies have consistently validated that aberrant accumulation of free fatty acids within the lipotoxic microenvironment of obesity acts as a pivotal inducer of defective efferocytosis. Among all fatty acid subtypes, palmitic acid (PA) represents the best-characterized metabolic stress signal in investigations linking obesity to impaired efferocytosis. Macrophages residing in distinct tissues serve as the primary phagocytes responsible for efferocytosis and orchestrating obesity-associated inflammatory disorders, and their functional abnormalities directly dictate the resolution trajectory of local inflammation, with representative subtypes including bone marrow-derived macrophages, alveolar macrophages, and colonic lamina propria macrophages (**Figure 3**).

PA triggers multi-dimensional defects in macrophage efferocytosis through three distinct mechanisms: regulation of kinase signaling cascades, epigenetic remodeling, and cellular metabolic reprogramming. Specifically, *in vitro* experiments have verified that PA exposure markedly suppresses AMPK activity in macrophages, which subsequently activates the mTORC1-ROCK1 signaling axis. This cascade directly impairs the capacity of macrophages to recognize and engulf apoptotic cells (81), yet the *in vivo* regulatory efficacy of this mechanism remains to be further validated. In addition, PA remodels active enhancer regions marked by histone H3K27ac in human monocyte-derived macrophages. This epigenetic alteration not only upregulates the transcription of pro-inflammatory genes including IRAK2 and IL-6, but also represses the expression of MerTK, thereby persistently blocking apoptotic cell clearance (82). *In vivo* studies conducted in obese mice further demonstrate that histone H3K4me3 modification broadly represses the transcription of glycolysis-related genes in bone marrow-derived macrophages. This transcriptional inhibition leads to insufficient energy supply and subsequent impairment of efferocytosis (83). However, this work only establishes a correlative relationship among aberrant epigenetic modifications, metabolic dysfunction and defective efferocytosis under obese conditions, targeted intervention assays are still lacking to precisely confirm their upstream-downstream causal regulatory hierarchy.

Notably, macrophages distributed across distinct tissues display prominent tissue-specific alterations in efferocytosis under obese conditions. Clinical data reveal that the phagocytic index of alveolar macrophages in obese patients complicated with asthma is reduced by approximately 40% relative to healthy controls, accompanied by concurrent downregulation of anti-inflammatory macrophage markers including PPARδ and CX3CR1 (84). Moreover, obesity drives macrophages toward a pro-inflammatory polarization prior to their infiltration into bronchial mucosa (85). In stark contrast, obesity exerts a protective regulatory effect on colonic lamina propria macrophages. The colon of high-fat diet (HFD)-induced obese mice is enriched with a lamina propria macrophage subset featuring elevated expression of phagocytosis-related genes. This cellular population sustains intestinal immune homeostasis and alleviates low-grade intestinal inflammation triggered by obesity (86). It should be noted that these observations are currently limited to murine models, and whether species differences interfere with the tissue-specific protective effects on efferocytosis remains unclear.

Based on the above mechanisms underlying obesity-associated efferocytic dysfunction, the development of AMPK agonists and selective mTORC1 inhibitors is expected to reverse PA-induced efferocytosis suppression and accelerate the resolution of obesity-related systemic inflammation. Furthermore, the core mechanisms driving the divergent functional phenotypes of airway and intestinal macrophages in obesity have not been fully elucidated. Subsequent studies can focus on theoretical hypotheses including gut microbial metabolites, tissue embryonic origins, and tissue-specific transcriptional regulatory networks to further explore the tissue-specific functional differentiation of macrophages.

Obesity impairs macrophage efferocytosis via two core pathways: 1. Cytosolic signaling axes: PA inhibits AMPK activity, activates ROCK1 and upregulates mTORC1 signaling; the expression of EPOR was downregulating in obesity state, ultimately significantly reducing macrophage phagocytic capacity. 2. Nuclear epigenetic reprogramming: PA induces histone H3K27ac reshaping, thus upregulates pro-inflammatory genes and downregulates phagocytic genes; obesity state also downregulates glycolysis-related genes via suppressing histone H3K4me3 in macrophage. Additionally, impaired macrophage efferocytosis exhibits tissue heterogeneity in obesity: airway macrophages show a marked decrease in phagocytic index and pro-inflammatory phenotype activation, while specific subsets of colonic lamina propria macrophages maintain phagocytosis-associated gene expression to preserve intestinal immune homeostasis.

### 4.2 DM and its microvascular complications

**Figure 4:**
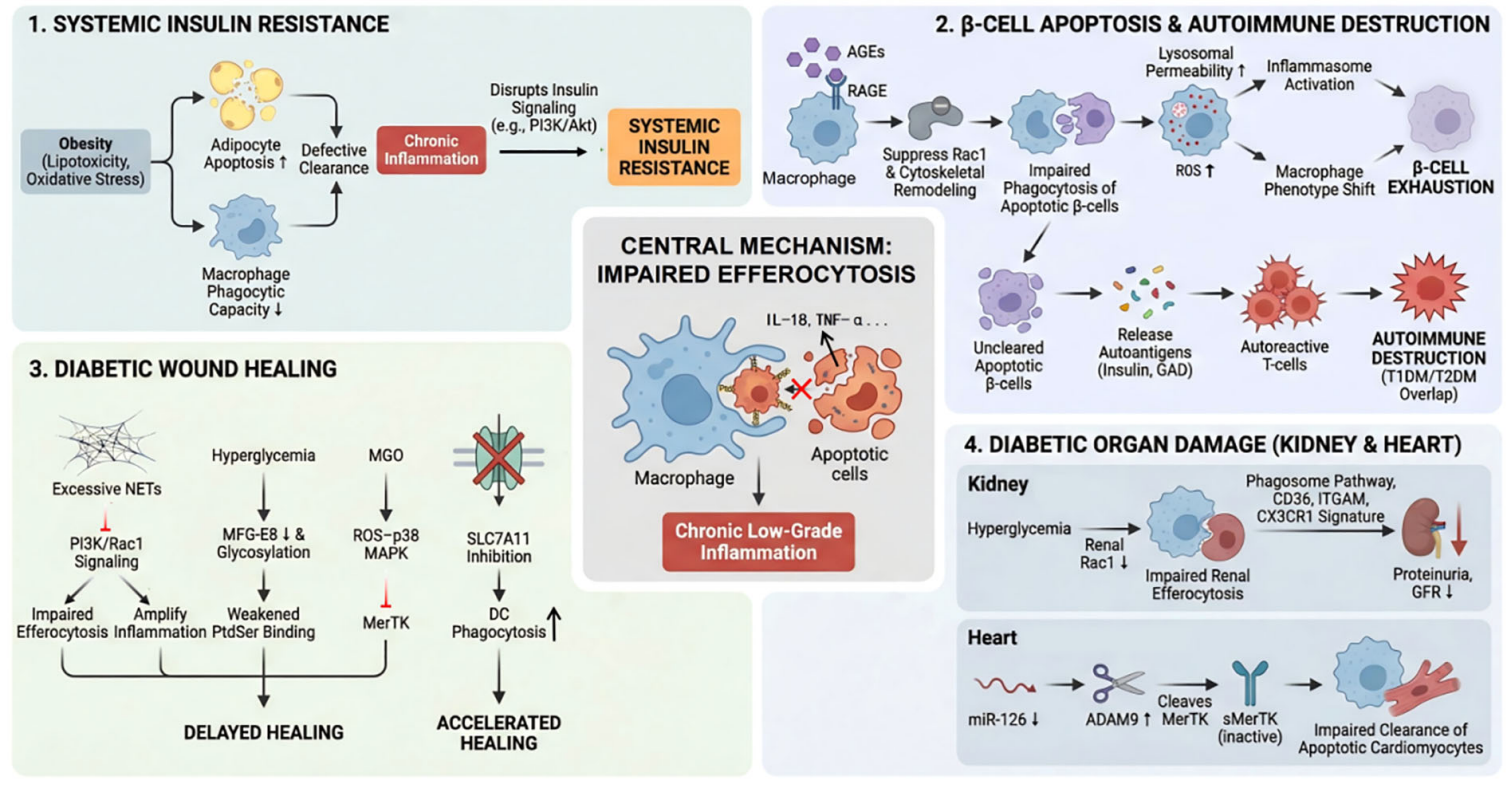
Pathological mechanisms of impaired efferocytosis in DM and its microvascular complications.

DM encompasses a spectrum of metabolic disorders characterized by impaired insulin secretion or defective insulin signaling, with type 1 diabetes mellitus (T1DM) and type 2 diabetes mellitus (T2DM) as the two predominant clinical subtypes (87). During the progression of DM, hyperglycemia, dyslipidemia, AGEs, and metaflammation act collectively as upstream stressors to synergistically impair the physiological regulatory network governing efferocytosis (88). Such efferocytic dysfunction not only profoundly accelerates diabetic progression, but also triggers and aggravates a wide range of diabetic microvascular complications (89, 90) (**Figure 4**).

Deficient efferocytic function correlates closely with DM onset and worsening complications, with the central mechanism being defective clearance of apoptotic cells by macrophages, which triggers chronic low-grade inflammation. Obesity-related lipotoxicity and oxidative stress increase adipocyte apoptosis and reduce macrophage phagocytic capacity. Defective clearance induces chronic inflammation, which further exacerbates insulin resistance. The AGEs-RAGE pathway inhibits macrophage Rac1 activity, leading to impaired clearance of apoptotic β-cells. Unremoved cells undergo secondary necrosis, release autoantigens, trigger autoimmune exhaustion of β-cell. Hyperglycemia inhibits MFG-E8 expression and weakens PtdSer binding via MFG-E8 glycosylation, and downregulates MerTK through the ROS-p38 MAPK pathway. In addition, SLC7A11 inhibition further impairs dendritic cell phagocytosis, ultimately leading to delayed wound healing. In the kidney, hyperglycemia inhibits Rac1 activity, impairs renal macrophage efferocytosis, and induces proteinuria and renal function decline. In the state of DM, the downregulation of miR-126 in the heart will activate the ADAM9 protein, which cleaves MerTK to generate sMerTK, leading to impaired clearance of apoptotic cardiomyocytes.

#### 4.2.1 DM

Impaired efferocytosis is tightly linked to progressive deterioration of insulin secretion. Stimulation by hyperglycemia, lipotoxicity and accumulated AGEs triggers massive apoptosis of pancreatic β-cells (91, 92). Resident pancreatic macrophages serve as the primary phagocytes responsible for clearing apoptotic β-cells. AGEs bind to the receptor for advanced glycation end products (RAGE) on pancreatic macrophages, which in turn suppresses Rac1 activity, blocks cytoskeletal remodeling in macrophages, and directly impairs their capacity to recognize and engulf apoptotic cells. Defective efferocytic clearance leads to persistent accumulation of apoptotic β-cells, further exacerbating chronic inflammation within the pancreatic microenvironment and accelerating β-cell exhaustion (93). Notably, *in vitro* co-culture assays have demonstrated that upon engulfing apoptotic β-cells, macrophages undergo pathological alterations including elevated lysosomal membrane permeability and robust intracellular ROS accumulation. These changes drive pro-inflammatory reprogramming of macrophages, which further disrupts pancreatic islet functional homeostasis (94). Nevertheless, this study fails to incorporate combined stimulation of upstream stressors (hyperglycemia, AGEs and lipotoxicity). In addition, it lacks validation using human pancreatic tissues, multiple diabetic animal models, and rescue experiments with gene editing, resulting in suboptimal reliability of its evidence.

Local islet efferocytosis imbalance not only exacerbates pancreatic inflammation but also triggers tissue-specific secondary autoimmune disturbance. During the progression of T2DM, defective efferocytosis leads to persistent retention of apoptotic β-cells in pancreatic islets. These cells constitutively secrete β-cell-specific autoantigens, namely insulin and glutamic acid decarboxylase (GAD). Persistent exposure to these autoantigens activates autoreactive T lymphocytes and initiates autoimmune destruction of pancreatic β-cells (95). Accordingly, targeting efferocytosis may contribute to constrain secondary autoimmune responses and attenuate phenotypic overlap as well as transitional progression between T1DM and T2DM. Nevertheless, this pathological cascade is currently only inferred from preclinical experiments, and large-scale clinical cohort studies are still absent to validate its actual contribution to subtype transition in diabetic populations.

#### 4.2.2 Diabetic wound

Chronic refractory diabetic wounds constitute an urgent clinical hurdle among diabetic microvascular complications (96). The core mechanism underlying persistent non-healing wounds is tightly associated with locally disrupted macrophage efferocytosis and impaired inflammation resolution within wound microenvironments, which are synergistically driven by glucose, its associated metabolites and neutrophil extracellular traps (NETs).

Emerging evidence reveals that abundant NETs accumulated in diabetic wounds suppress the PI3K/Rac1 signaling cascade, hamper cytoskeletal remodeling in macrophages, further block the internalization of apoptotic cells, and thereby directly reduce the local efferocytic clearance capacity of wounds (97). However, this study only adopted STZ-induced T1DM mice, while the vast majority of intractable diabetic foot ulcers observed clinically occur in patients with T2DM. Accordingly, the reliability of translating these findings to T2DM wounds remains limited. Other investigations have demonstrated that a hyperglycemic microenvironment markedly downregulates the expression of MFG-E8 in granulation tissues of diabetic wounds and triggers its glycosylation modification. Such post-translational modifications drastically weaken the binding affinity between MFG-E8 and PtdSer, ultimately delaying wound re-epithelialization and restraining angiogenesis (98). Hence, the expression profile of MFG-E8 in diabetic wound tissue has the potential to serve as a promising biomarker for predicting the risk of persistent chronic ulceration in diabetic wounds.

Methylglyoxal (MGO), a highly reactive glucose metabolite abundantly accumulated under diabetic conditions, acts as a critical metabolic mediator driving defective efferocytosis in wounds. MGO activates the ROS-p38 MAPK signaling axis to downregulate MerTK expression in macrophages, impairs macrophage recognition of apoptotic cells, disrupts wound efferocytic homeostasis, and sustains a pro-inflammatory microenvironment within wound sites (99). Regrettably, this regulatory mechanism has only been validated in preclinical studies. No clinical investigations have confirmed correlations between MGO concentrations in peripheral circulation or wound exudate and actual wound repair prognosis. Accordingly, its clinical value as a biomarker for wound stratification and therapeutic response prediction remains to be verified.

Solute carrier family 7 member 11 (SLC7A11) serves as the master regulatory molecule governing cysteine-glutamate transport (100). Emerging evidence indicates that inhibition of SLC7A11 activity in dendritic cells (DCs) enhances their phagocytic capacity toward apoptotic cells and accelerates wound repair in diabetic lesions. Nevertheless, SLC7A11 suppression exerts dual bidirectional effects: while boosting efferocytosis, it concurrently triggers cellular ferroptosis (101, 102). Beyond the SLC7A11-dependent regulatory axis, multilayered bidirectional crosstalk circuits exist between ferroptosis and efferocytosis. For instance, preliminary evidence indicates that oxidized phospholipids released by ferroptotic cells initiate efferocytic cascades via the macrophage CD36 receptor. Conversely, reactive oxygen species and oxidized lipids secreted by apoptotic cells reciprocally activate ferroptotic signaling, exacerbating local oxidative stress injury (103). Future investigations are required to further elucidate the dynamic balance and spatiotemporal regulatory patterns linking ferroptosis and efferocytosis within the diabetic wound microenvironment.

#### 4.2.3 Diabetic kidney disease

Beyond cutaneous wound injury, diabetic kidney disease (DKD) represents another prevalent and prognostically poor microvascular complication of DM, and dysregulated efferocytosis is speculated to participate in its chronic pathological progression. High-throughput sequencing have revealed that renal macrophage populations undergo continuous dynamic remodeling during DKD progression, with the phagosome pathway identified as the most significantly enriched functional pathway in renal lesions (86). This study further screened three core hub genes closely associated with renal efferocytic homeostasis, namely CD36, ITGAM, and CX3CR1, whose expression fluctuations are strongly correlated with proteinuria severity and progressive renal function decline in patients. These findings suggest that defective macrophage efferocytosis may serve as a core pathological regulatory mechanism underlying DKD.

Nevertheless, current conclusions are primarily derived from bulk transcriptomic enrichment analyses, which only indicate correlations between differential gene expression and disease phenotypes, rather than fully elucidating the region-specific functions of these three core genes and other efferocytosis-related molecules in distinct anatomical compartments of the kidney. The renal glomeruli, tubulointerstitium, and perivascular regions possess distinct microenvironmental characteristics, and macrophages exhibit obvious spatial heterogeneity in infiltration abundance, activation status, and efferocytic capacity across different renal regions. Therefore, future studies combining spatial transcriptomics with *in vivo* and *in vitro* functional validation experiments are required to systematically dissect the region-specific expression patterns and functional differences of efferocytosis-related genes, so as to precisely clarify the refined mechanisms by which macrophage efferocytosis dysfunction mediates renal injury in DKD.

#### 4.2.4 Diabetic cardiomyopathy

Diabetic cardiomyopathy (DCM) is an independent metabolic myocardial lesion that develops in diabetic patients without other primary cardiac diseases (104). Dysfunction of efferocytosis may serves as a vital mechanism mediating chronic myocardial injury and progressive cardiac dysfunction. Accumulating emerging evidence indicates that a high-glucose microenvironment markedly downregulates miR-126 expression in bone marrow-derived macrophages from db/db mice and human THP-1 cells, which further upregulates the metalloprotease ADAM9. Elevated ADAM9 proteolytically cleaves MerTK, the core efferocytic receptor on the cell membrane, to generate soluble sMerTK devoid of biological function. This process effectively blocks downstream phagocytic signaling cascades and contributes to the pathological progression of DCM (105). Members of the metalloprotease family exhibit overlapping functions, yet current studies only focus on ADAM9-mediated MerTK cleavage. It remains unclear whether other homologous metalloproteases such as ADAM17 participate in this modification process, which limits comprehensive understanding of the regulatory network underlying disrupted efferocytosis in DCM. Given the crucial roles of miR-126 and sMerTK in regulating efferocytosis, their circulating peripheral expression levels have the potential to indirectly reflect the systemic efferocytic status of DCM patients, offering novel research insights for early warning, dynamic disease monitoring and individualized therapeutic efficacy evaluation of DCM.

### 4.3 MASLD

**Figure 5:**
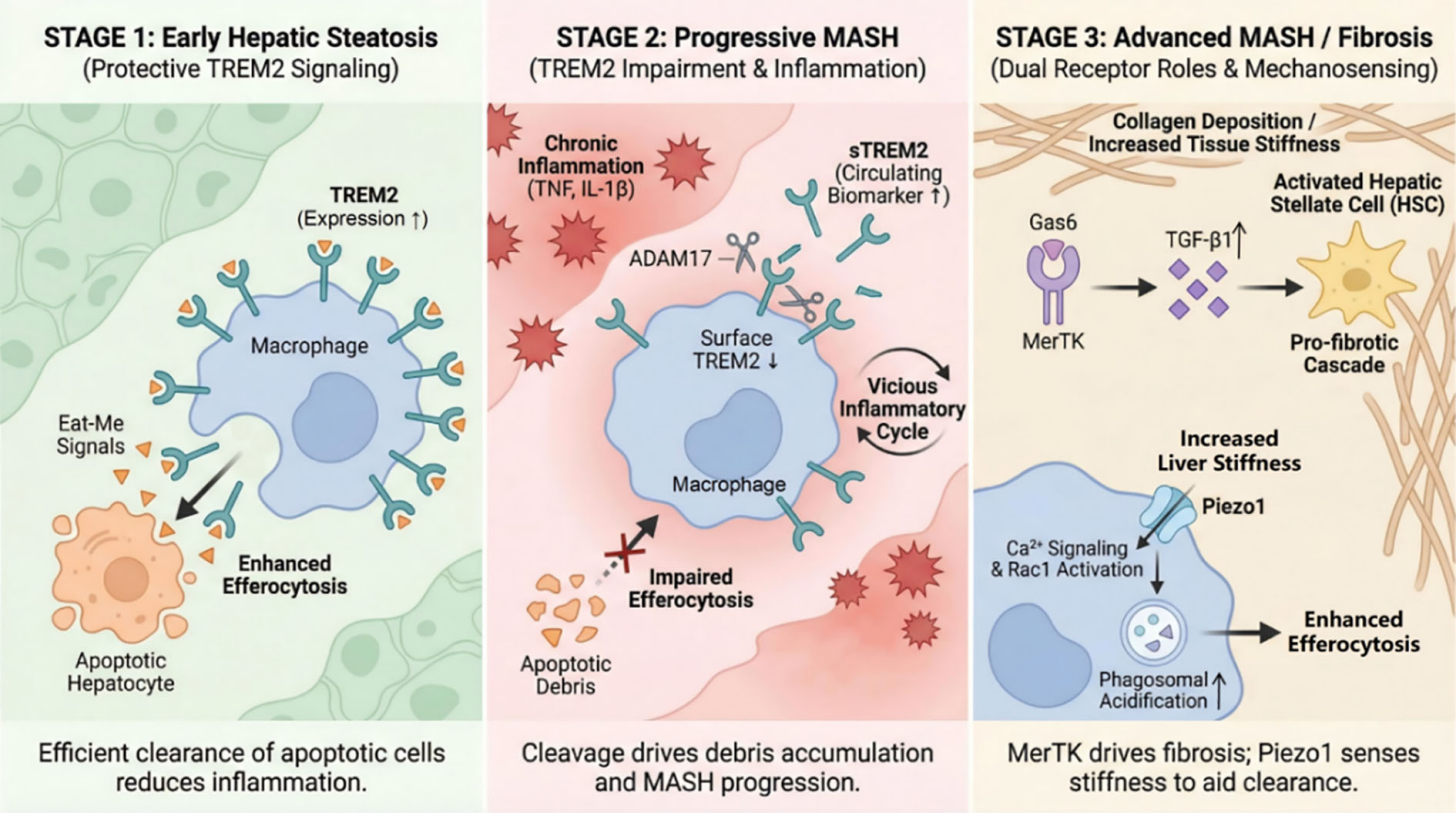
Efferocytosis regulation during different stages of MASLD.

MASLD, previously termed non-alcoholic fatty liver disease (NAFLD), is a highly prevalent metabolic liver disease worldwide, with a population prevalence exceeding 30% (106, 107). MASLD features a progressive and continuous pathological spectrum, which can advance from isolated hepatic steatosis to metabolic dysfunction-associated steatohepatitis (MASH), and further trigger hepatic fibrosis, cirrhosis and even hepatocellular carcinoma. It is a critical target organ lesion of the liver resulting from multi-organ injury induced by metabolic disorders (108). Lipotoxic accumulation and persistent chronic inflammation act as core upstream metabolic stressors that disrupt hepatic microenvironment homeostasis, impair efferocytosis, and drive stepwise deterioration of MASLD (**Figure 5**) (109).

The core phagocytes involved in efferocytosis regulation and mediating pathological progression of MASLD in the liver mainly consist of two populations: resident hepatic Kupffer cells and peripherally infiltrating monocyte-derived macrophages (110). Both subsets of hepatic macrophages possess strong functional plasticity and undergo phenotypic and functional remodeling in response to dynamic shifts in hepatic metabolic and inflammatory microenvironments, serving as the central cellular regulators that determine the inflammatory outcome of MASLD.

Disordered efferocytosis during MASLD progression displays prominent tissue- and disease stage-specific features. In terms of tissue specificity, abnormally elevated tissue stiffness accompanying liver fibrosis specifically activates the Piezo1 mechanosensory signaling pathway. By regulating phagosome acidification in macrophages and the clearance efficiency of apoptotic hepatocytes, this pathway constructs a liver-specific mechanosensitive efferocytosis regulatory circuit (111). With regard to disease stage specificity, the functions of core efferocytic receptors undergo dynamic transformation alongside disease progression. TREM2 is compensatorily upregulated in the early simple hepatic steatosis stage of MASLD. It exerts hepatoprotective effects by amplifying efferocytic signaling to timely eliminate damaged hepatocytes (112–114). As the disease advances to MASH and fibrotic stages, the locally enriched pro-inflammatory microenvironment triggers ADAM17-mediated proteolytic cleavage and ectodomain shedding of surface TREM2 on macrophages. This leads to the loss of functional TREM2, persistent impairment of hepatic efferocytosis, and sustained unresolved inflammatory injury in the liver (115).

The MerTK pathway exhibits functional characteristics entirely opposite to those of TREM2 throughout the progression of MASLD, forming a dual molecular framework governing hepatic efferocytosis. Persistent dysregulated lipid metabolism in MASLD aberrantly activates the Gas6-dependent MerTK signaling cascade, which drives robust secretion of TGF-β1 in macrophages. This subsequently sustains activation of fibrogenic pathways in hepatic stellate cells, triggers abnormal hepatic collagen deposition, and accelerates the advancement of liver fibrosis (116). Clinical studies have demonstrated that MASLD patients carrying loss-of-function MerTK mutations face a markedly reduced risk of aggravated liver fibrosis, verifying that aberrant MerTK activation acts as a critical molecular trigger for disrupted hepatic efferocytosis and fibrotic injury (117). Such functional shift of MerTK during chronic metabolic liver injury represents a prominent feature in current research on efferocytosis in MASLD, and its precise upstream regulatory mechanisms remain to be further elucidated in detail.

Based on the above research evidence, soluble TREM2 (sTREM2), a circulating peripheral product generated via proteolytic shedding of TREM2, may indirectly reflect the severity of hepatic efferocytic defects and the level of inflammatory activity. It holds promise as a non-invasive molecular biomarker for stratifying MASLD severity, staging disease progression, evaluating therapeutic responses and screening high-risk populations (118, 119), yet its diagnostic specificity and sensitivity require systematic verification in large-scale clinical cohorts. Meanwhile, the MerTK pathway exerts dual functions of anti-inflammatory repair and fibrogenesis promotion. Developing temporally and region-specific targeted intervention strategies that precisely match the progressive pathological microenvironment of MASLD to block the pathogenic effects of MerTK represents a critical research direction for realizing precision therapy against MASLD.

In early hepatic steatosis stage, macrophages highly express TREM2, which significantly enhances efferocytosis to efficiently clear apoptotic hepatocytes, exerting anti-inflammatory and protective effects. In progressive MASH stage, chronic inflammation activates ADAM17, which cleaves TREM2 to generate soluble sTREM2 and impaired efferocytosis. Accumulation of apoptotic debris forms a vicious inflammatory cycle, accelerating the progression of MASLD. In advanced MASH stage, MerTK activates hepatic stellate cells via the Gas6-TGF-β1 axis and triggering its pro-fibrotic cascade. While the mechanosensor Piezo1 senses increased tissue stiffness, promotes phagosomal acidification through Ca²^+^-dependent activation of Rac1, enhances apoptotic hepatocyte clearance, and mediates a protective response.

### 4.4 Atherosclerosis

Atherosclerosis is a metabolic cardiovascular disease tightly associated with obesity and DM. Destabilization of atherosclerotic plaques formed in the wall of large arteries serves as the core pathological basis for major adverse cardiovascular events (120). Abnormal accumulation of ox-LDL within plaques, persistent hyperglycemic stimulation and the local chronic pro-inflammatory microenvironment collectively construct a multidimensional metabolic stress system. This system leads to the buildup of apoptotic cells and cellular debris, which drives persistent plaque inflammation and lesion progression.

Macrophages act as professional phagocytes that regulate efferocytosis and mediate inflammation resolution within the atherosclerotic plaque microenvironment, and their functional homeostasis directly governs the inflammatory status of plaque microenvironment as well as plaque stability (18). In addition, vascular smooth muscle cells and vascular endothelial cells are key non-professional phagocytes participating in plaque efferocytosis modulation. Relying on their inherent cellular plasticity, these two cell types engage in local vascular debris clearance and pathological vascular remodeling (121).

Multiple metabolic stressors within atherosclerotic plaques trigger aberrant alterations in efferocytic function of the aforementioned phagocytes. For instance, the chronic pro-inflammatory microenvironment inside plaques activates matrix metalloproteinase-dependent proteolysis, which specifically cleaves MerTK (122). Meanwhile, necroptotic cells induced by TNF signaling within plaques overexpress the Don’t-Eat-Me” signal CD47 abnormally (123, 124). Disturbed lipid and glucose metabolism in plaques also hinder efferocytosis at multiple steps. ox-LDL competitively antagonizes the “Eat-Me” signal PtdSer exposed on apoptotic cells to block the initial recognition stage of efferocytosis, and simultaneously suppresses actin polymerization in macrophages (125, 126). High-glucose microenvironment downregulates miR-369-3p expression in plaque macrophages, repressing mitochondrial respiratory function and phagocytic activity. It also boosts robust secretion of pro-inflammatory cytokines, which further amplifies local inflammatory injury within plaques (127, 128).

**Figure 6:**
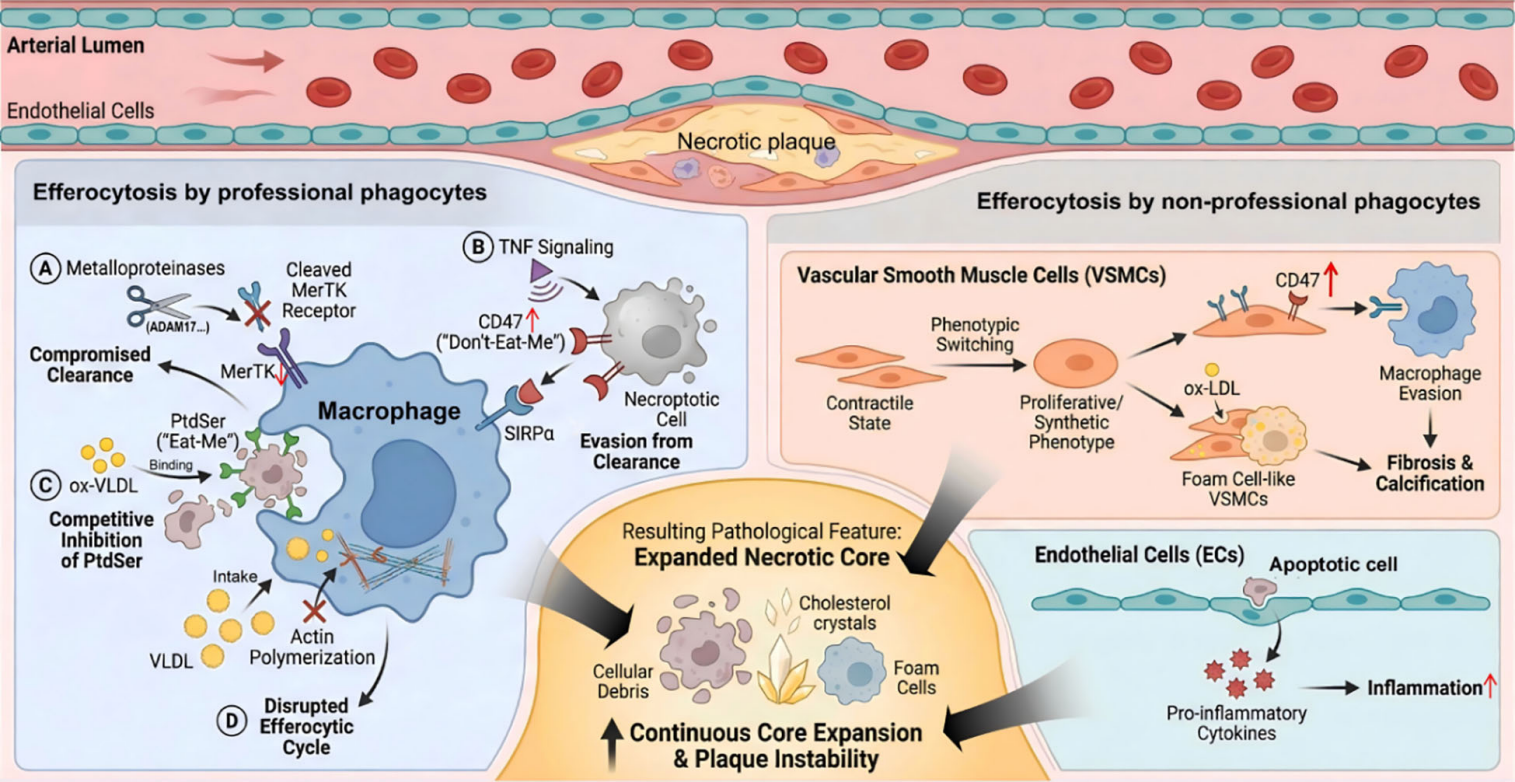
Efferocytosis driven by professional and non-professional phagocytes in atherosclerosis.

In contrast to the compromised efferocytic function of macrophages induced by diverse metabolic stressors, efferocytic behaviors of non-professional phagocytes display distinct bidirectional functional phenotypes throughout atherosclerotic development (129). Emerging evidence reveals that vascular smooth muscle cells are capable of engulfing ox-LDL within plaques, facilitating foam cell generation, progressively enlarging the plaque necrotic core (130, 131). By comparison, upon engulfing apoptotic cells, vascular endothelial cells fail to trigger anti-inflammatory and tissue-reparative cascades; instead, they boost the secretion of pro-inflammatory cytokines and aggravate local inflammatory damage (132) (**Figure 6**). Collectively, these two subsets of non-professional phagocytes—vascular smooth muscle cells and vascular endothelial cells—appear incapable of mediating robust anti-inflammatory responses and tissue repair.

Collectively, existing studies suggest that modulating mitochondrial function in macrophages and blocking the antagonistic effect of ox-LDL on efferocytic pathways hold promise for rescuing metabolic stress-induced efferocytic dysfunction and halting plaque progression. Notably, current evidence supporting the dual functions of non-professional phagocytes including vascular smooth muscle cells and vascular endothelial cells is exclusively derived from *in vitro* cellular experiments. Their functional phenotypes, regulatory mechanisms and relative contribution within the complex *in vivo* plaque microenvironment have not been systematically validated. This constitutes a critical knowledge gap that needs to be addressed in efferocytosis research focusing on non-professional phagocytes, as well as a key research direction for further deciphering the pathological mechanisms underlying atherosclerosis.

Preclinical evidence indicates that defective efferocytosis may serve as an amplifying pathogenic mechanism driving the transition of atherosclerotic plaques from stable to vulnerable phenotypes, involving dual functional abnormalities of professional and non-professional phagocytes. In professional phagocytes (macrophages): 1. Metalloproteinases such as ADAM17 cleave MerTK receptors, disrupting the recognition of PtdSer-mediated “Eat-Me” signals; 2. TNF signaling upregulates the CD47-SIRPα “Don’t-Eat-Me” axis, leading to evasion of necrotic cells from clearance; 3. ox-VLDL competitively inhibits “Eat-Me” signal PtdSer; 4. VLDL interferes with actin polymerization, disrupting efferocytic cycle. In non-professional phagocytes: Vascular smooth muscle cells undergo phenotypic switching, and ox-LDL induces their foam cell-like transformation with upregulated CD47, evading macrophage clearance and promoting fibrosis and calcification; The phagocytosis of apoptotic cells by endothelial cells releases pro-inflammatory cytokines, which exacerbates the local inflammatory response.

## 5 Intervention strategies targeting efferocytosis for metabolic disorders

As outlined above, efferocytosis occupies a central position in the pathogenesis of multiple metabolic disorders. Developing intervention strategies to mitigate chronic persistent inflammation in metabolic disorders via enhancing efferocytosis has accordingly emerged as a thriving direction in translational medical research. Relevant approaches span a broad spectrum ranging from broad-spectrum small-molecule agents and highly specific monoclonal antibodies to sophisticated nanoparticle delivery systems alongside lifestyle-modulating regimens.

### 5.1 Clinically available drugs

PPARγ is one of the key metabolite-sensing receptors on efferocytic phagocytes, which acts in concert with core efferocytic receptors such as MerTK to orchestrate the clearance of apoptotic cells by phagocytes (133). Agonists targeting the PPARγ in phagocytes exhibit prominent potential in preclinical models of metabolic disorders. These agents effectively mimic the lipid metabolic responses triggered by apoptotic cell engulfment, upregulate the expression of anti-inflammatory factors and the engulfment receptor in phagocytes. For instance, EPO enhances macrophage efferocytosis via a PPARγ-dependent mechanism, thereby alleviating chronic inflammation in obese mice (134). Thus, the beneficial effects of EPO in patients with end-stage DKD may extend beyond the correction of anemia, and it may also confer renoprotective effects by enhancing efferocytosis. However, in non-anemic individuals, EPO elevates erythrocyte counts and increases thrombotic risk, limiting its suitability for long-term anti-inflammatory intervention. Future research may prioritize structural modification of EPO to develop optimized peptides or small molecules that retain PPARγ-activating capacity while avoiding activation of the EPOR mediated hematopoietic pathway. Additionally, exploring clinically available agonists targeting lipid sensing nuclear receptors for their potential to rescue defective efferocytosis in metabolic disorders holds considerable translational significance. Representative agents include the anti-diabetic PPARγ agonists thiazolidinediones (TZDs) or the PPARα/γ/δ triple agonist chiglitazar, the PPARδ agonist seladelpar for primary biliary cholangitis, and the broad-spectrum PPARα/δ/γ triple agonist bezafibrate used in hyperlipidemia.

Statins, including atorvastatin and rosuvastatin, as cornerstone lipid-lowering agents for cardiovascular disorders also exert pro-efferocytic effect (135). By inhibiting the mevalonate metabolic pathway, statins modulate small GTPase activity in macrophages, suppressing the RhoA-mediated anti-phagocytic pathway while activating the Rac1-driven pro-phagocytic cascade. This further regulates actin polymerization membrane ruffling and phagocytic cup formation ultimately enhancing macrophage-mediated clearance of apoptotic cells (136). Besides that, statins boost efferocytosis by restricting nuclear translocation of NF-κB1 and downregulating expression of the “Don’t-Eat-Me” signal CD47 (137). These findings indicate that statins may confer additional protective benefits against obesity, MASLD and atherosclerosis via their pro-efferocytic properties. Remarkably, long-term or high-dose statin administration may correlate with adverse reactions including myotoxicity, hepatic injury, and neurocognitive disorder (138–140). Accordingly, comparative analyses across different statins remain necessary to screen optimal agents integrating potent lipid-lowering efficacy with robust pro-efferocytic activity, and to define their optimal therapeutic dosages. Further investigations are also required to clarify whether the pro-efferocytic effects of statins exhibit specificity toward macrophage subsets and dependence on the tissue microenvironment.

### 5.2 Synthetic or natural compounds

Beyond drug repurposing strategies, synthetic compounds also exert promising therapeutic effects against metabolic disorders by enhancing efferocytosis. For instance, 4-octyl itaconate (4-OI) upregulates monocarboxylate transporter 1 (MCT1) expression in macrophages at diabetic wound sites, promotes lactate release during efferocytosis, and further activates the GPR132 receptor, ultimately driving macrophage polarization toward an anti-inflammatory phenotype (141). Although this finding provides novel insights and directions for diabetic wound therapy by targeting lactate metabolism, diabetic wounds are characterized by disorganized collagen tissue and edematous compression. Moreover, the poor permeability of free 4-OI may impede its efficient accumulation in macrophage-enriched regions of deep wound tissues (142, 143). Therefore, further optimization of 4-OI drug delivery systems is warranted to improve its tissue permeability and bioavailability. Additionally, it remains necessary to validate whether 4-OI regulates efferocytosis via upregulating lactylation modifications of efferocytosis-related receptors or bridging molecules, or by modulating the expression of core efferocytosis genes through histone lactylation.

Similar to the synthetic metabolic regulatory small molecule 4-OI, plant-derived natural compounds can also restore efferocytic function under metabolic disturbance, with the additional advantages of higher safety and easier availability. As a common active component in traditional Chinese medicines (e.g., Astragalus membranaceus, Bupleurum chinense), quercetin has been validated by network pharmacology studies to exert potential therapeutic effects on obesity-related Hashimoto’s thyroiditis by promoting macrophage efferocytosis (144–146). However, the regulation of efferocytosis by quercetin lacks direct experimental evidence, the “false positive targets” identified via network pharmacology have not been excluded. In addition, sulforaphane contained in cruciferous vegetables has been shown to accelerate wound healing in diabetic mice by upregulating MerTK expression in macrophages (147). Isoliquiritigenin, a flavonoid compound mainly derived from the rhizomes of Glycyrrhiza uralensis, can enhance the phagocytic capacity and glycolytic activity of DCs by inhibiting SLC7A11 in diabetic wounds (148). However, it remains necessary to further explore whether isoliquiritigenin exerts any negative impact on the antigen-presenting function of DCs, so as to clarify its comprehensive pharmacological effects.

### 5.3 Targeted monoclonal antibodies

Compared with small-molecule compounds lacking tissue or cell specificity, monoclonal antibodies can precisely target cell-surface proteins, offering a highly specific and efficient approach for regulating efferocytosis. Among these, the most extensively investigated strategy with broad clinical potential is the use of specific antibodies to block the “Don’t-Eat-Me” signal CD47. Healthy cells express CD47, which binds to the SIRPα receptor on phagocytes and transmits inhibitory “Don’t-Eat-Me” signals to evade phagocytosis (149). While apoptotic cells in atherosclerotic plaque can abnormally upregulate CD47 to evade immune clearance (150, 151). A retrospective analysis of clinical trial data for the anti-CD47 antibody magrolimab demonstrated reduced inflammation in atherosclerotic lesions among treated patients, thereby providing robust clinical evidence supporting CD47 as a viable therapeutic target for alleviating atherosclerosis (152, 153). Given that efferocytic dysfunction represents a common pathological mechanism underlying multiple metabolic disorders, the therapeutic efficacy of anti-CD47 antibody remains to be further validated in metabolic disorders beyond atherosclerosis. In addition to CD47, other immune checkpoints, such as CD24 and CD31, have also been shown to mediate efferocytic escape (71, 154). Nevertheless, the actual clinical translational value of against these cell surface markers for metabolic diseases remains to be evaluated.

### 5.4 Nanomaterials and targeted delivery systems

Most functional proteins or hydrophobic small molecules with pro-efferocytic activity face considerable limitations for *in vivo* application, including poor targeting specificity, short half-life, and low retention efficiency. In contrast, surface-functionalized nanocarriers enable precise tissue targeting and even cell-type-specific delivery of these agents, as well as stimulus-responsive release in the metabolic stress microenvironment, thereby significantly reducing systemic toxicity (155, 156). In addition, nano drug delivery systems fabricated from novel delivery materials can further improve drug stability, prolong systemic circulation time, and enhance drug accumulation at lesion sites via rational encapsulation and controlled-release design (157). These nanocarriers and drug delivery systems provide promising strategies for addressing impaired efferocytosis in metabolic disorders.

For example, the hybrid membrane nanovesicles constructed by Shuo Qiu et al. can target and deliver functional MerTK to plaque macrophages, markedly delaying the progression of atherosclerosis in diabetic mice without disrupting systemic immune homeostasis (158). Beyond this, MiQu et al. designed drug-loaded extracellular vesicles (EVs) targeting VCAM1 and modified the EVs surface with PtdSer to provide an “Eat-Me” signal, thereby promoting apoptosis and efferocytic clearance of senescent cells in diabetic mice (159, 160). In another two studies, PtdSer-decorated liposomes encapsulating an insulin peptide antigen were constructed, which induced the generation of tolerogenic DCs, thus achieving antigen-specific immune tolerance toward pancreatic β-cells in T1DM (161). On the basis of the aforementioned researches, further development of multifunctional integrated nanocarriers (such as those loaded with the extracellular domain of MerTK and anti-CD47 antibodies) will provide critical support for clinical translation of efferocytosis-regulating strategies in metabolic disorders.

Although advanced nanomaterial-based delivery platforms engineered for metabolic disorders hold prominent potential for clinical translation, their practical application still encounters several essential issues. On the one hand, the biocompatibility and long-term *in vivo* safety of these nanomaterials require more systematic evaluation and validation, so as to minimize risks related to organ accumulation and potential adverse immune responses. On the other hand, inconsistent and non-standardized laboratory preparation protocols fail to satisfy the rigorous demands for standardization and batch-to-batch consistency during large-scale manufacturing (162, 163).

### 5.5 Lifestyle interventions

In the clinical management of metabolic disorders, fundamental interventions such as dietary modification and physical exercise remain indispensable. These strategies reflect the multidimensional and systematic concepts of metabolic disorders management, are easily implemented in clinical practice, and demonstrate high patient acceptability. Although accumulating studies have demonstrated that such lifestyle interventions are generally safe and can markedly improve efferocytic function, all mechanistic evidence is derived from rodent models. Further validation via human experimental research and clinical cohort data is therefore warranted.

#### 5.5.1 Dietary interventions

Among various lipid components, n-3 PUFAs are supported by the most conclusive evidence for their pro-phagocytic effects. Recent study demonstrated that supplementation with fish oil rich in n-3 fatty acids in obese ob/ob mice restored macrophage membrane fluidity by improving the lipid composition of cell membranes and normalizing the PI3K/AKT signaling pathway—both of which are essential for efficient phagocytosis (164). This finding provides a novel mechanistic basis for the well-established anti-inflammatory and cardiovascular protective effects of n-3 PUFAs. However, the underlying reasons why different types of fatty acids (e.g., PA and PUFAs) exert completely opposite effects on efferocytosis remain to be further explored from multiple dimensions, including downstream signaling cascades, metabolic pathways, epigenetic regulation, and gut microbiota-mediated regulation. Along with this, quercetin — a flavonoid abundantly present in common fruits and vegetables such as onions, broccoli, cherries, and grapes — has been verified via network pharmacology exhibits strong binding affinity to targets including CD36, and MerTK (145). It should be emphasized that such computational results only provide preliminary theoretical clues indicating that flavonoid-rich dietary patterns may help sustain the homeostasis of peripheral macrophages. Cellular or animal-based functional validation experiments are still required to confirm this regulatory phenotype.

#### 5.5.2 Exercise interventions

Exercise is a crucial cornerstone for maintaining metabolic health. Studies have confirmed that aerobic exercise training can upregulate the expression level of macrophage lysosomal protease, thereby enhancing the phagocytic clearance capacity of macrophages for apoptotic cardiomyocytes (165). This helps alleviate pathological myocardial remodeling in metabolic-related cardiovascular diseases such as diabetic cardiomyopathy and atherosclerotic heart disease. Developmental endothelial locus-1 (DEL-1) is an exercise-induced myokine primarily secreted by vascular endothelial cells and skeletal muscle cells. As reported by Kwon et al., exogenous administration of recombinant DEL-1 can effectively attenuate inflammatory and improve insulin resistance in differentiated 3T3-L1 adipocytes (166). Given that HO-1 has been confirmed to upregulate the expression of the efferocytic receptor MerTK in macrophages (167), it is reasonable to hypothesize that exercise may enhance the efferocytic function of adipose tissue macrophages by elevating DEL-1 levels, thereby mitigating insulin resistance caused by systemic inflammation. Comparing the effects of different exercise intensities and durations on the secretion of various myokines including DEL-1 and irisin, and exploring whether these myokines exert regulatory effects on efferocytosis will help prevent secondary injuries caused by excessive exercise in patients with metabolic disorders.

Of note, studies have demonstrated that even in the absence of physical activity, distal muscle neuromuscular electrical stimulation can enhance macrophage efferocytosis and accelerate wound healing by upregulating the expression of midkine in muscles (168). This innovative exercise-mimetic therapy provides a noninvasive, home-operable, and dose-controllable pro-efferocytic treatment strategy for diabetic foot patients at high amputation risk who are unable to perform regular physical exercise. However, it is noteworthy that repeated electrical stimulation may induce potential nerve damage in diabetic patients with peripheral neuropathy. Therefore, a comprehensive evaluation of the clinical safety of this intervention is necessary.

## 6 Controversies and translational challenges

Efferocytosis serves as a core immune regulatory mechanism that maintains metabolic homeostasis through sophisticated signaling networks. Although targeted modulation of efferocytosis holds promising therapeutic potential for metabolic disorders such as obesity, MASLD, DM, and atherosclerosis, the inherent complexity of its regulatory pathways gives rise to multiple controversies and translational bottlenecks in advancing from preclinical mechanistic research to clinical practice.

### 6.1 The functional duality of efferocytosis

Although enhancing efferocytosis represents a well-defined therapeutic strategy for alleviating chronic inflammation in metabolic disorders, tumor cells can exploit this mechanism to mediate immune evasion and accelerate systemic metastasis. The core mechanism lies in the fact that the phagocytosis of apoptotic tumor cells by tumor-associated macrophages (TAMs) promotes the secretion of anti-inflammatory cytokines and the expansion of regulatory T cells (Tregs), thereby establishing an immunosuppressive tumor microenvironment that impairs the efficient clearance of apoptotic tumor cells by TAMs (169, 170). Based on this immunosuppressive mechanism, strategies targeting efferocytosis inhibition are under development in the field of oncology, including antibodies targeting PtdSer (e.g., bavituximab) and inhibitors of TAM family receptors (171, 172). While these anti-efferocytic agents exert positive effects in cancer treatment, they are a “double-edged sword” that may aggravate metabolic stress injury caused by comorbidities like DM and obesity. This dual effect clearly indicates that the potential adverse impacts of such anti-tumor agents on the overall efferocytic homeostasis of patients require rigorous and systematic evaluation. The use of these agents must be individually tailored according to the patient’s disease spectrum and specific clinical context.

### 6.2 Specificity of CD47-targeted antibodies

Blocking the CD47-mediated “Don’t-Eat-Me” signal serves as an effective strategy to enhance the clearance of apoptotic cells accumulated in metabolic disorders such as atherosclerosis. However, CD47 is ubiquitously expressed on the surface of healthy cells, including erythrocytes and platelets, and systemic CD47 blockade therefore readily induces off-target hematological toxicities, such as anemia and hemorrhage (173–175). Owing to these severe hematological adverse events, the phase III ENHANCE clinical trial of the CD47 monoclonal antibody magrolimab for high-risk myelodysplastic syndrome and acute myeloid leukemia was terminated in July 2023 (176), prompting extensive re-evaluation of the clinical feasibility of systemic CD47-targeting strategies.

Although emerging nanocarrier delivery systems provide a promising approach to alleviate the off-target damage of CD47-targeted antibodies, their clinical translation faces substantial challenges beyond conventional manufacturing and basic safety concerns. Insufficient circulatory stability and premature drug release severely impede the spatiotemporal precision of drug delivery (177); interindividual heterogeneity of the physiological microenvironment reduces the reproducibility of targeting efficiency and therapeutic outcomes (178); furthermore, the formation of a protein corona on nanocarriers after blood exposure reshapes their *in vivo* biodistribution and compromises their specific targeting capacity toward pathological lesions (179).

### 6.3 Dynamic monitoring and quantification of efferocytosis

Studies on efferocytic clearance mechanisms in metabolic disorders remain constrained by numerous methodological limitations. Current research approaches rely on static ex vivo tissue section analysis (such as immunofluorescence colocalization and electron microscopy) or indirect detection of downstream anti-inflammatory effects and transcriptomic to monitor changes in efferocytic function(**Table 1**). These methods only capture transient snapshots of the highly dynamic apoptotic cell clearance process including recognition, internalization, and degradation. They are inadequate for accurate, real-time, dynamic quantification of efferocytosis *in vivo*, particularly in deep tissues such as the pancreas and liver (187). Genetically encoded reporter mice with macrophage membrane-targeted expression of pH-sensitive pHluorin, combined with *in vivo* two-photon microscopy, may enable long-term real-time visual observation of dynamic efferocytic behavior in living tissues by tracking the characteristic fluorescence quenching signals within internalized acidified phagosomes.

**Table 1:** Summary of detection methods related to efferocytosis.

Methods	Principle	Parameters	Specimens	Advantages	Disadvantages	Reliability	Ref.
Electron microscopy	The high resolution of electron microscopy allows morphological confirmation of efferocytosis	The morphology and quantity of apoptotic bodies within phagosomes, as well as the structural characteristics of phagosomes and phagolysosomes	①Cell samples; ②Tissue samples	Ultra-high resolution, serving as the morphological gold standard for efferocytosis	extremely low throughput, providing only static snapshots; sample preparation may easily cause specimen damage	Extremely high	(180) (181),
Flow cytometry	Phagocytes are labeled with fluorescently conjugated antibodies, and apoptotic target cells are labeled with membrane-permeable or pH-sensitive fluorescent dyes. Following co-culture, efferocytosis efficiency is quantified	Efferocytosis index; number and percentage of double-positive cells	living cells *in vitro*	High-throughput, enabling rapid acquisition of large datasets; stable and reproducible results; allows quantitative analysis of efferocytosis efficiency with relatively simple operation	Lacks direct visual evidence of phagocytosis and cannot distinguish between binding of apoptotic cells to the cell surface and complete internalization. Moreover, it is difficult to reflect spatial interactions between cells.	High	(182) (183),
Cellular fluorescence microscopy	Phagocytes and apoptotic cells are fluorescently labeled, and their interactions are observed under a microscope. The CharON dual-fluorescent probe system allows real-time tracking of the efferocytosis process and distinguishes between surface-bound and internalized apoptotic cells	Colocalization of phagocytes and apoptotic cells	living cells *in vitro*	Provides direct visual evidence of phagocytosis, presents the spatial distribution and dynamic process of efferocytosis, distinguishes surface binding from complete internalization	Low throughput, quantitative results prone to subjective bias, and unable to reflect dynamic processes	High	(167)
*In situ* immunofluorescence	Immunofluorescence staining of tissue sections was performed to verify efferocytosis efficiency via colocalization of phagocyte markers and apoptotic markers	The colocalization ratio of phagocyte markers (F4/80, CD68) and apoptotic markers (TUNEL, cleaved caspase-3)	Tissue samples	It can reflect the actual status of efferocytosis in the physiological microenvironment and provide direct evidence of *in vivo* efferocytosis, and can clear identification of the distribution characteristics of efferocytosis within tissues	High cost, extremely demanding for experimental animals and operational skills; low throughput, quantitative results prone to subjective bias, and unable to reflect dynamic processes	High	(184)
Omics Technologies	scRNA-seq is used to dissect phagocyte heterogeneity and efferocytosis-related macrophage subsets. Spatial transcriptomics preserves the anatomical context of efferocytosis-associated gene expression. Metabolomics and stable isotope tracing are employed to track efferocytosis-related metabolic reprogramming	Macrophage subsets and differentially expressed genes; spatial expression profiles of efferocytosis-related genes; the types and contents of efferocytosis-associated metabolites	①Cell samples; ②Tissue samples	It can reveal the regulatory mechanisms of efferocytosis at the molecular, genetic, and metabolic levels, identify novel efferocytosis-associated molecular targets and metabolic pathways, and dissect the heterogeneity of efferocytosis	Extremely high cost; difficult data analysis and inability to directly quantify efferocytosis efficiency	High	(73)
Seahorse XF analysis	Based on the metabolic reprogramming characteristics of phagocytes during efferocytosis, indicators related to mitochondrial oxidative phosphorylation and glycolysis are detected to reflect the metabolic requirements of efferocytosis	① Oxygen consumption rate (OCR); ②Extracellular acidification rate (ECAR)	①Living cells *in vitro*; ②Phagocytes isolated *in vivo*	Real-time detection of metabolic dynamics during the efferocytosis process	High cost; cannot directly reflect phagocytic behavior, and the sample processing procedure is complicated	Moderate high	(78)
Efferocytosis biomarkers detection	Based on the cleavage and release of key surface receptors on phagocytes by metalloproteinases or altered expression of downstream anti-inflammatory cytokines during dysfunctional efferocytosis, the efferocytic status can be reflected by detecting soluble biomarkers	① Circulating levels of sMerTK and sTREM2; ②Expression levels of anti-inflammatory mediators (IL-10 and TGF-β); ③Expression levels of efferocytosis-related functional molecules such as MerTK, Gas6, ABCA1, etc.	①Serum; ②Tissue homogenates; ③Cell culture supernatants; ④Cell lysates	Non-invasive or minimally invasive detection of *in vivo* efferocytic function is feasible, suitable for clinical translation and large-scale screening. The procedure is relatively simple and allows quantitative analysis	Limited specificity; some biomarkers may be influenced by other inflammatory or physiological processes. Cannot directly reflect phagocytic activity and only serves as an indirect indicator. Detection methods for certain biomarkers are not yet standardized	Moderate	(185) (186),

Additionally, the absence of standardized, clinically validated, and noninvasive biomarkers for monitoring efferocytic efficiency in humans constitutes another crucial bottleneck that hinders the efficacy evaluation of efferocytic targeted therapies. Although soluble receptors such as sMerTK and sTREM2 exhibit potential as candidate biomarkers, their levels are susceptible to interference from systemic inflammatory responses and matrix metalloproteinase activity (such as AMAM9/17) *in vivo*. This susceptibility can lead to biased interpretations when these soluble receptors are adopted as specific indicators of efferocytic dysfunction (188, 189). In addition, indices such as the “efferocytic index” vary considerably depending on the cell type used, the timing of detection, and the calculation method employed, rendering direct comparisons across different studies challenging.

## 7 Conclusion and future perspectives

**Figure 7:**
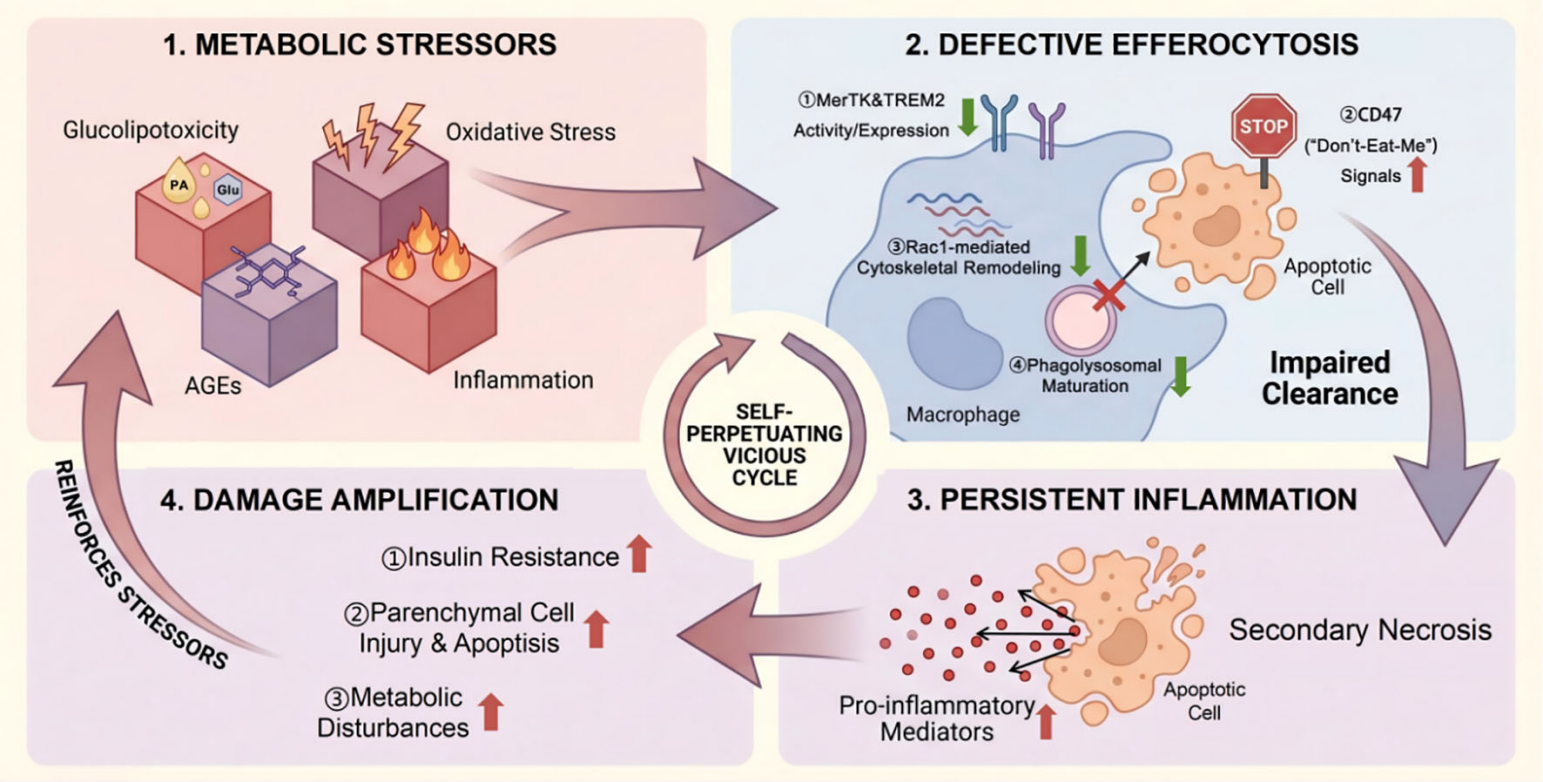
The unified mechanism governing the vicious cycle of “metabolic stress-impaired efferocytosis-inflammatory amplification” across distinct metabolic disorders.

Accumulating preclinical data further implies that impaired efferocytosis may serve as a unified pathogenic amplifier connecting sustained inflammatory signaling and exacerbating disease progression across metabolic disorders. Specifically, glucolipotoxicity, oxidative stress, AGEs, NETs and chronic inflammation associated with metabolic disorders directly impair macrophage efferocytosis and induce defective apoptotic cell clearance. This impairment is achieved by downregulating the expression and activity of efferocytic receptors (e.g., MerTK and TREM2) (127, 153), disrupting Rac1-mediated cytoskeletal remodeling (77, 101), upregulating “Don’t-Eat-Me” signals (e.g., CD47) on apoptotic cells (73, 143, 158), and inducing phagolysosomal dysfunction (122). Uncleared apoptotic cells undergo secondary necrosis and release abundant pro-inflammatory mediators, which further amplify chronic low-grade tissue inflammation. Sustained excessive inflammation, in turn, exacerbates insulin resistance, parenchymal cell injury and apoptosis, aggravates metabolic disorders, and ultimately forms a vicious feedback cycle (103, 148) (**Figure 7**).

In metabolic disorders, metabolic stressors including glucolipotoxicity, AGEs, oxidative stress, and inflammation induce defective macrophage efferocytosis through multiple mechanisms: downregulated expression/activity of key efferocytosis receptors such as MerTK and TREM2, upregulated “Don’t Eat Me” signal CD47, impaired Rac1-mediated cytoskeletal remodeling, and blocked phagolysosomal maturation, ultimately leading to impaired apoptotic cell clearance. Unremoved apoptotic cells undergo secondary necrosis, releasing pro-inflammatory mediators and triggering persistent inflammation. This further exacerbates insulin resistance, parenchymal cell injury and apoptosis, and metabolic disturbances, reinforcing the effects of metabolic stressors and forming a mutually reinforcing, self-perpetuating vicious cycle in diverse metabolic disorders.

Future studies should focus on exploring the association and underlying molecular mechanisms between efferocytic dysfunction and special metabolic disease subtypes, including gestational DM, pediatric metabolic disorders, and sarcopenic obesity. In addition, sex hormones and divergent adipose immune microenvironments between males and females may shape distinct efferocytic phenotypes of phagocytes, dedicated preclinical and clinical investigations are urgently warranted to clarify sex-based disparities in efferocytic dysfunction across metabolic disorders. Finally, clarifying whether metabolic stress states induce persistent epigenetic metabolic memory in phagocytes and their progenitors and thereby impair efferocytosis represents another key research direction.

In summary, we believe that further in-depth dissection of the complex regulatory networks associated with efferocytosis in metabolic disorders will enable us to establish innovative clinical paradigms for the early diagnosis and prevention of these diseases, opening new avenues for comprehensive management of metabolic disorders progression incorporating pharmacotherapy, diet, and exercise. Ultimately, this will provide more precise and effective medical strategies for billions of patients with metabolic disorders and significantly improve their long-term prognosis.

## Glossary

ABCA1: ATP-binding cassette transporter A1
ADAM9: A disintegrin and metalloproteinase domain-containing protein 9
AGEs: Advanced glycation end products
APOE: Apolipoprotein E
APOJ: Apolipoprotein J
ATP: Adenosine triphosphate
BM-MSCs: Bone marrow mesenchymal stem cells
CX3CL1: Chemokine C-X3-C motif ligand 1
DAMPs: Damage-associated molecular patterns
DCM: Diabetic cardiomyopathy
DCs: Dendritic cells
DEL-1: Developmental endothelial locus-1
DKD: Diabetic kidney disease
DM: Diabetes mellitus
ECAR: Extracellular acidification rate
EPOR: Erythropoietin receptor
FFAs: Free fatty acids
GBD: Global Burden of Disease
GEF: Guanine nucleotide exchange factor
GFR: Glomerular filtration rate
HFD: High fat diet
IDF: International Diabetes Federation
LPA: Lysophosphatidic acid
LPC: Lysophosphatidylcholine
LRP1: Low-density lipoprotein receptor-related protein 1
MASLD: Metabolic dysfunction-associated steatotic liver disease
MASH: Metabolic dysfunction-associated steatohepatitis
MCT1: Monocarboxylate transporter 1
MGO: Methylglyoxal
MSCs: Mesenchymal stem cells
NAFLD: Non-alcoholic fatty liver disease
NETs: Neutrophil extracellular traps
NSAIDs: Nonsteroidal anti-inflammatory drugs
OCR: Oxygen consumption rate
ox-LDL: Oxidized low-density lipoprotein
ox-VLDL: Oxidized very-low-density lipoprotein
PA: Palmitic acid
PANX1: Pannexin 1
PtdSer: Phosphatidylserine
PUFAs: Polyunsaturated fatty acids
RAGE: Receptor for advanced glycation end products
ROS: Reactive oxygen species
sMerTK: Soluble MerTK
sTREM2: Soluble TREM2
SIRPα: Signal regulatory protein α
S1P: Sphingosine 1-phosphate
T1DM: Type 1 diabetes mellitus
T2DM: Type 2 diabetes mellitus
TNF: Tumor necrosis factor
TZDs: Thiazolidinediones
UTP: Uridine triphosphate
4-OI: 4-Octyl itaconate.
